# Disulfide Dimerization of Neuronal Calcium Sensor-1: Implications for Zinc and Redox Signaling

**DOI:** 10.3390/ijms222212602

**Published:** 2021-11-22

**Authors:** Viktoriia E. Baksheeva, Alexey V. Baldin, Arthur O. Zalevsky, Aliya A. Nazipova, Alexey S. Kazakov, Vasiliy I. Vladimirov, Neonila V. Gorokhovets, François Devred, Pavel P. Philippov, Alexandr V. Bazhin, Andrey V. Golovin, Andrey A. Zamyatnin, Dmitry V. Zinchenko, Philipp O. Tsvetkov, Sergei E. Permyakov, Evgeni Yu. Zernii

**Affiliations:** 1 Belozersky Institute of Physico Chemical Biology, Lomonosov Moscow State University, 119992 Moscow, Russia; vbaksheeva@belozersky.msu.ru (V.E.B.); alexeyvbaldin@gmail.com (A.V.B.); ppph@belozersky.msu.ru (P.P.P.); zamyat@belozersky.msu.ru (A.A.Z.J.); 2 Department of General, Visceral, and Transplant Surgery, Ludwig Maximilians University Munich, 81377 Munich, Germany; alexandr.bazhin@uni-muenchen.de; 3 V.I. Kulakov National Medical Research Center of Obstetrics, Gynecology and Perinatology, 117997 Moscow, Russia; 4 Shemyakin Ovchinnikov Institute of Bioorganic Chemistry of the Russian Academy of Sciences, 117997 Moscow, Russia; aozalevsky@fbb.msu.ru (A.O.Z.); vladimirov@bibch.ru (V.I.V.); golovin@fbb.msu.ru (A.V.G.); zdv@bibch.ru (D.V.Z.); 5 Institute for Biological Instrumentation, Pushchino Scientific Center for Biological Research of the Russian Academy of Sciences, 142290 Pushchino, Russia; alija-alex@rambler.ru (A.A.N.); fenixfly@yandex.ru (A.S.K.); permyakov.s@gmail.com (S.E.P.); 6 Institute of Molecular Medicine, Sechenov First Moscow State Medical University, 119991 Moscow, Russia; gorokhovets_n_v@staff.sechenov.ru; 7 Institut de Neurophysiopathologie, INP, CNRS, Faculté des Sciences Médicales et Paramédicales, Aix-Marseille Université, 13005 Marseille, France; francois.devred@univ-amu.fr (F.D.); philipp.tsvetkov@univ-amu.fr (P.O.T.); 8 Plateforme Interactome Timone, PINT, Faculté des Sciences Médicales et Paramédicales, Aix Marseille Université, 13009 Marseille, France; 9 Partner Site Munich, German Cancer Consortium (DKTK), 80336 Munich, Germany; 10 Faculty of Bioengineering and Bioinformatics, Lomonosov Moscow State University, 119234 Moscow, Russia; 11 Sirius University of Science and Technology, 354340 Sochi, Russia; 12 Faculty of Health and Medical Sciences, University of Surrey, Guildford GU2 7XH, Surrey, UK

**Keywords:** EF-hand, NCS family, neuronal calcium sensor-1, disulfide dimerization, GRK1, zinc, protein aggregation, apoptosis, neurodegeneration, cancer

## Abstract

Neuronal calcium sensor-1 (NCS-1) is a four-EF-hand ubiquitous signaling protein modulating neuronal function and survival, which participates in neurodegeneration and carcinogenesis. NCS-1 recognizes specific sites on cellular membranes and regulates numerous targets, including G-protein coupled receptors and their kinases (GRKs). Here, with the use of cellular models and various biophysical and computational techniques, we demonstrate that NCS-1 is a redox-sensitive protein, which responds to oxidizing conditions by the formation of disulfide dimer (dNCS-1), involving its single, highly conservative cysteine C38. The dimer content is unaffected by the elevation of intracellular calcium levels but increases to 10–30% at high free zinc concentrations (characteristic of oxidative stress), which is accompanied by accumulation of the protein in punctual clusters in the perinuclear area. The formation of dNCS-1 represents a specific Zn^2+^-promoted process, requiring proper folding of the protein and occurring at redox potential values approaching apoptotic levels. The dimer binds Ca^2+^ only in one EF-hand per monomer, thereby representing a unique state, with decreased α-helicity and thermal stability, increased surface hydrophobicity, and markedly improved inhibitory activity against GRK1 due to 20-fold higher affinity towards the enzyme. Furthermore, dNCS-1 can coordinate zinc and, according to molecular modeling, has an asymmetrical structure and increased conformational flexibility of the subunits, which may underlie their enhanced target-binding properties. In HEK293 cells, dNCS-1 can be reduced by the thioredoxin system, otherwise accumulating as protein aggregates, which are degraded by the proteasome. Interestingly, NCS-1 silencing diminishes the susceptibility of Y79 cancer cells to oxidative stress-induced apoptosis, suggesting that NCS-1 may mediate redox-regulated pathways governing cell death/survival in response to oxidative conditions.

## 1. Introduction

Neuronal calcium sensors (NCSs) are a family of signaling proteins expressed mainly in neurons and retinal photoreceptors and are involved in the regulation of various aspects of neuronal function in response to calcium signals. NCSs are characterized by a similar two-domain organization, with four EF-hand-type Ca^2+^-binding motifs (EF1 is always non-functional) and a myristoyl group at the N-terminus (in some NCSs, a palmitoyl group is attached to cysteine residues in the N-terminal domain) (reviewed in [1,2]). Some of these proteins exhibit constitutive membrane association, while others employ a Ca^2+^-myristoyl switch mechanism, i.e., reversible Ca^2+^-induced exposure of the myristoyl group, ensuring binding to membranes and compartmentalization with target proteins [3,4,5,6,7,8]. Consistently, the majority of these targets are transmembrane or membrane-associated proteins, such as cell surface receptors, ion channels, effector enzymes, and others (reviewed in [9]).

Neuronal calcium sensor-1 (NCS-1) is the ancestral member of the NCS family. It possesses relatively high Ca^2+^-binding affinity (K_D_ ~ 0.3 μM) and is constitutively associated with plasma or Golgi membranes, preferring sites enriched in negatively charged phospholipids, such as phosphatidylserine or signaling phosphatidylinositol phosphates [10,11,12]. NCS-1 has three functional EF-hand motifs, which can coordinate magnesium and display different affinity and filling orders for calcium: EF2 and EF3 are occupied first, followed by the occupation of EF4 [13]. NCS-1 binds and regulates more than 20 protein targets, including other Ca^2+^-binding proteins, potassium and calcium channels, and phosphatidylinositol kinases, as well as various metabotropic receptors and associated signaling proteins, including G-protein coupled receptors (GPCRs) and G-protein coupled receptor kinases (GRKs) (reviewed in [14,15,16,17]). Due to these properties, it participates in the regulation of neurotransmission, cell growth and survival, and synaptic plasticity underlying mechanisms of learning and memory. Multiple studies have implicated NCS-1 in the regulation of sensory systems, such as thermotaxis in *Caenorhabditis elegans* and olfactory pathways and visual transduction in mammals [8,18,19,20,21]. Recently, NCS-1 has been found to interact with and regulate TRPV4, a transient receptor potential channel transducing various nociceptive responses and mediating neuropathic pain, induced, for instance, by chemotherapy agent paclitaxel [22]. Abnormalities in the expression and function of NCS-1, as well as hereditary mutations in its gene, are associated with neurodegenerative and neuropsychiatric diseases, including schizophrenia, autism, Alzheimer’s disease, and Parkinson’s disease [23]. In addition, NCS-1 is aberrantly expressed in certain cancers, and the level of its expression correlates with tumor aggressiveness and patients’ survival rates [24,25].

Previous studies have demonstrated that some NCSs, such as visinine-like protein-1 (VILIP-1) and recoverin, are redox-sensitive proteins that form disulfide dimers (and apparently cysteine oxidized monomers [26,27,28,29]) in response to an increase in the oxidative potential of the intercellular medium [27,28,29,30,31,32,33]. Disulfide dimerization of VILIP-1 involves its unique C-terminal C187 and affects the interaction of the protein with physiological targets, such as guanylate cyclase B. VILIP-1 dimers are accumulated in soluble or aggregated form in the spinal cord of patients with amyotrophic lateral sclerosis (ALS), thereby representing a hallmark of the disease [30,31,32]. In the case of recoverin, disulfide dimerization is provided by oxidation of its single C39, located in the first, non-functional EF-hand motif. Disulfide dimer of recoverin exhibits altered structure and functional properties, such as improved membrane binding and regulatory activity towards GRK1 in the absence of Ca^2+^. It accumulates in mammalian retinas exposed to intense light irradiation, imitating oxidative conditions associated with age-related macular degeneration (AMD) [27,28,33]. Thus, oxidation of NCS proteins may contribute to the mechanisms triggered in response to oxidative stress, leading to neuronal degeneration. NCS-1 also contains single cysteine C38 (homologous to C39 in recoverin), which is a highly conserved residue among NCS proteins. An early study addressing structural and functional properties of recombinant NCS-1 demonstrated that C38 changes surface accessibility upon Ca^2+^ binding, suggesting the possible participation of the protein in redox signaling [34]. Consistently, several studies report that NCS-1 may play a role in cell survival under conditions of oxidative stress [35,36,37].

Notably, oxidative stress is commonly associated with the release of intracellular zinc (increased concentrations of so-called loosely-bound zinc), high levels of which are normally contained in neuronal tissue in a protein-bound form [38]. Zinc is released mainly from metallothionein buffer proteins in response to the oxidation of cysteine residues in their zinc finger sites [39]. Elevation of zinc concentrations produces neurotoxicity associated with multiple neurological disorders, including Alzheimer’s disease, AMD, and glaucoma [40,41,42]. The effects of loosely-bound zinc are mediated by regulatory proteins containing various binding sites for this metal (reviewed in [38]). The conditions of oxidative stress are also recognized by specialized proteins triggering mechanisms of redox regulation and compensatory responses in cells [43,44]. Previously, we have demonstrated that recoverin can combine these functional features, being both a redox-sensitive and zinc-binding protein [27,28,33,45]. NCS-1 is also capable of binding zinc in both high-affinity and low-affinity sites, which modulates its functional status. High-affinity binding increases thermal stability and favors interaction with target proteins, whereas low-affinity binding promotes aggregation of the protein [12].

Considering these findings, the aims of the current study were as follows: to characterize the redox sensitivity of NCS-1 and evaluate the effect of zinc on this feature; to determine alterations in the structure and cellular activity of the protein under oxidizing conditions, and to assess the contribution of NCS-1 to zinc/redox-dependent mechanisms governing cell survival.

## 2. Results

### 2.1. Disulfide Dimerization of NCS-1 in Cells

**Figure 1:**
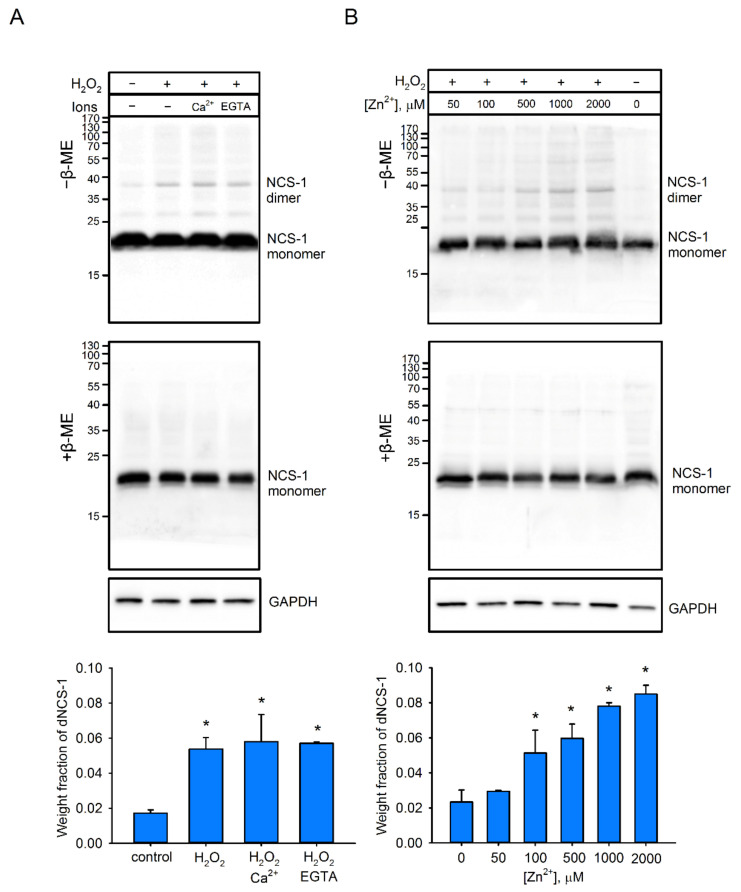
Disulfide dimerization of NCS-1 in HEK293 cells. (**A**) The cells transfected with NCS-1 were treated for 30 min with 10 mM H_2_O_2_ in the presence of calcium (and ionophore ionomycin) or its chelator (EGTA). Disulfide forms of NCS-1 were detected by Western blotting of cell lysates conducted under non-reducing (“−β-ME”) or reducing (“+β-ME”) conditions (**top**). Fractions of dNCS-1 were calculated densitometrically based on at least 3 independent experiments (**bottom**). * *p* < 0.05 as compared to dNCS-1 content in the absence of H_2_O_2_ treatment. (**B**) NCS-1-transfected cells were incubated for 30 min with 10 mM H_2_O_2_ and increasing concentrations of Zn^2+^ in the presence of zinc ionophore chloroquine. Fractions of dNCS-1 were calculated by densitometric analysis of Western blots of cell lysates (**top**) and plotted against Zn^2+^ concentrations (**bottom**). * *p* < 0.05 as compared to dNCS-1 content in the absence of zinc.

To assess the redox sensitivity of NCS-1 under cellular conditions, we employed HEK293 cells, in which moderate endogenous expression of this protein had been enhanced by transfection with a genetic construct, encoding the protein. Using this model, we found that induction of oxidative stress by 30 min of treatment with 10 mM H_2_O_2_ led to the formation of dimeric NCS-1. According to Western blotting under non-reducing conditions, the fraction of the dimer (~40 kDa) in cellular extracts reached about 4–5% of the total NCS-1, and this proportion did not change upon induction of Ca^2+^ influx into the cells, using ionomycin or chelation of calcium with EGTA (Figure 1A). Under reducing conditions, NCS-1 appeared as a single band, corresponding to monomeric protein (~23 kDa), indicating that the revealed dimer (dNCS-1) was stabilized by disulfide bonds. Electrophoretic mobility of dNCS1 in cell extracts exactly matched those of the disulfide dimer prepared from purified recombinant NCS-1 (Supplementary Figure S1; see Section 4.3 and Section 4.4). We also detected a few bands possibly representing disulfide complexes of NCS-1 with other proteins, but their fraction was less than 0.5%, indicating high specificity of the homodimerization. Notably, pretreatment of the cells with increasing concentrations of Zn^2+^ (in the presence of a permanent concentration of its ionophore chloroquine [46]) was found to gradually increase the fraction of dNCS-1, which reached ~9% of total protein (Figure 1B). Furthermore, prolongation of incubation of cells with Zn^2+^/H_2_O_2_ could increase dimer content up to 25–30% (Supplementary Figure S1). Thus, under oxidative stress conditions, NCS-1 can form a disulfide dimer, involving its single cysteine (C38), and this process is markedly stimulated with increased concentrations of intracellular zinc.

### 2.2. Recycling of dNCS-1 in Cells

**Figure 2:**
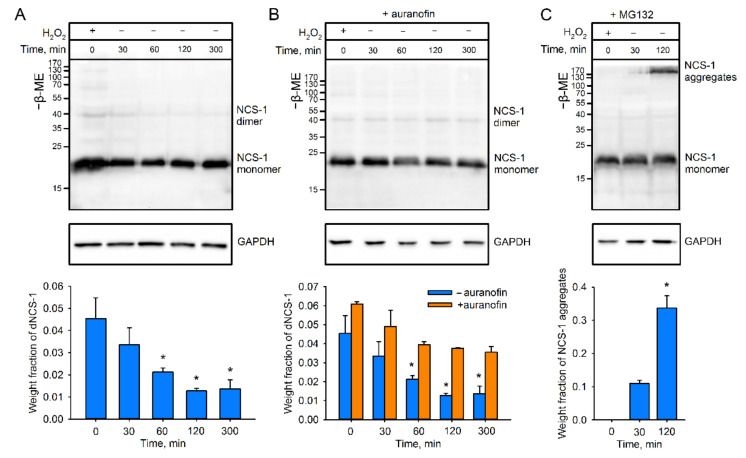
Mechanism of dNCS-1 recycling in HEK293 cells. (**A**) The cells transfected with NCS-1 were incubated in the presence of 10 mM H_2_O_2_ for 30 min and dNCS-1 content is monitored for the next 5 h of cultivation under non-oxidizing conditions. Relative dNCS-1 fractions (as compared to the content at the starting point) were calculated based on Western blotting of cell lysates (**top**) obtained in at least 3 independent experiments and plotted against time (**bottom**). * *p* < 0.05 as compared to dNCS-1 content at the starting point. (**B**,**C**) NCS-1-transfected cells pretreated with a thioredoxin reductase inhibitor auranofin (**B**) or proteasome inhibitor MG132 (C) were exposed to oxidative stress conditions and incubated as described in (**A**). Degradation of disulfide forms of NCS-1 was monitored by Western blotting of cell lysates (**top**) and their relative fractions (as compared to the content at the starting point) were plotted against time (**bottom**). * *p* < 0.05 as compared to dNCS-1 content in the starting point.

The described HEK293-based model was next employed to assess whether dNCS-1 can be spontaneously recovered in cells. It was found that disulfide dimerization of NCS-1 is indeed a reversible process. The decrease in cellular content of dNCS-1 started immediately after exposure to hydrogen peroxide, and during five-hour incubation in H_2_O_2_-free medium, the dimer became almost indistinguishable (Figure 2A). To unravel the potential mechanism underlying dNCS-1 utilization, we next investigated two possible directions of this process: reduction of the intermolecular disulfide bond by cellular antioxidant enzymes and degradation of the dimer by the proteasome [31]. Exposure of cells pretreated with thioredoxin reductase inhibitor auranofin dampened dNCS-1 utilization, indicating the involvement of the thioredoxin/thioredoxin reductase-dependent antioxidant system in reduction of the dimer (Figure 2B). Interestingly, suppression of proteasome activity using MG132 led to the formation of NCS-1-containing aggregates, the fraction of which was found to reach 50% of the total protein during the two hours following exposure (Figure 2C). The corresponding band was absent in blots prepared under reducing conditions, indicating that it corresponded to disulfide-stabilized forms (data not shown). Low amounts of dNCS-1 in these blots might be related to its efficient reduction due to compensatory stimulation of the antioxidant system in the case of proteasome inhibition [47]. We concluded that in cells, dNCS-1 can be reduced by mechanisms involving the thioredoxin/thioredoxin reductase system, whereas disulfide-dependent aggregates of the protein are degraded by the proteasome.

### 2.3. Cellular Localization of NCS-1 in Oxidative Stress

**Figure 3:**
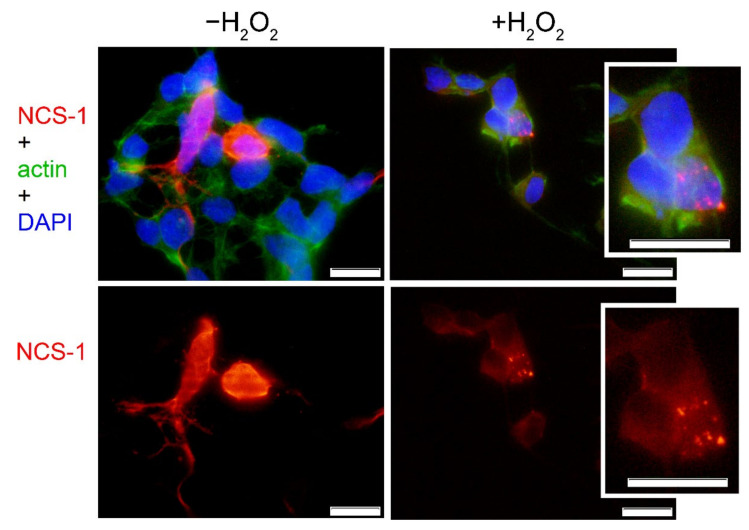
Localization of NCS-1 in HEK293 cells with or without oxidative stress. Representative microphotographs of NCS-1-trasfected cells, cultivated under normal (“−H_2_O_2_”) or oxidizing (“+H_2_O_2_”) conditions. NCS-1 was detected using polyclonal anti-NCS-1 primary antibodies and fluorescently labeled secondary antibodies. NCS-1, cytoskeleton (actin), and nuclei are stained in red, green, and blue colors, respectively. Scale bar: 1 μm.

Given the relatively high level of NCS-1 expression in the HEK293-based model, we also attempted to determine the patterns of its cellular localization and characterize oxidative stress-related changes in these patterns by means of fluorescence microscopy. Using antibodies against NCS-1, it was demonstrated that under normal conditions, the protein is distributed in the perinuclear region of HEK293 cells, which generally corresponds to its common localization in neurons and other cells (Figure 3) [48,49]. Meanwhile, under conditions of oxidative stress, we observed pronounced alterations in the distribution of the protein, with a significant fraction of NCS-1/dNCS-1 stained as punctual clusters around the nucleus. The nature of these clusters remained unclear, although we can propose that they represented oxidized protein recruited by autophagosomes or accumulated in aggresomes, forming insoluble deposits which are commonly associated with proteasome dysfunction (see Section 3, Discussion).

### 2.4. Factors Affecting dNCS-1 Formation

**Figure 4:**
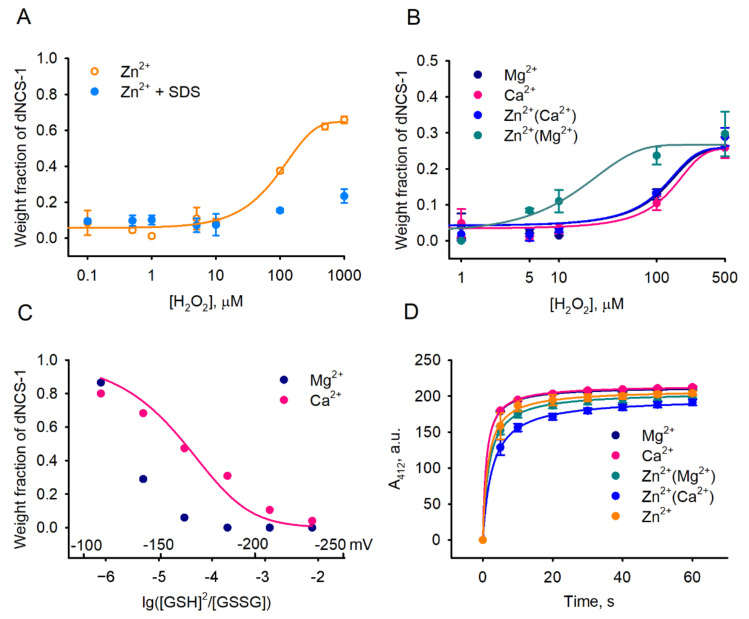
Disulfide dimerization of NCS-1 in vitro. (**A**,**B**) Disulfide dimerization of recombinant decalcified NCS-1 was induced by incubation with increasing concentrations of H_2_O_2_ in presence of 0.1 mM ZnCl_2_, 1% SDS, 1 mM CaCl_2_, 1 mM MgCl_2_ or their combinations. Weight fractions of dNCS-1 were determined by densitometric analysis of SDS-PAGE data in at least 3 independent experiments and plotted against H_2_O_2_ concentrations. (**C**) The transition between dNCS-1 and NCS-1 was monitored in presence of 0.1 mM GSSG and various concentrations of GSH (8.8 µM–72.4 mM). Weight fractions of dNCS-1 were calculated densitometrically from SDS-PAGE data and plotted against lg([GSH]^2^/[GSSG]). The corresponding values of redox potential were indicated on the *x*-axis. (**D**) Reactivity of SH-group (C38) of NCS-1 with DTNB (Ellman’s reagent) was assayed in the presence of 0.1 mM ZnCl_2_, 1 mM CaCl_2_, 1 mM MgCl_2_ or their combinations. Kinetics of the colorimetric reaction was monitored in at least 3 independent experiments by measuring the absorbance of TNB^2−^ at 412 nm.

To characterize, in more detail, the conditions favoring disulfide dimerization of NCS-1, we next examined the effect of various physiologically relevant factors on this process in a set of in vitro studies. It was found that treatment of recombinant myristoylated NCS-1 with increasing concentrations of hydrogen peroxide indeed promotes the formation of dNCS-1 (Figure 4A). Notably, disulfide dimerization was most pronounced in the case of Zn^2+^-bound NCS-1 obtained by incubation of apo-protein with zinc ions; in this case, dimer content could reach ~75%. Dimerization was almost completely inhibited in the presence of an ionic denaturing agent (SDS), indicating the dependence of this process on the proper folding of the protein. Given that, in cells, NCS-1 operates mainly as an Mg^2+^-bound or Ca^2+^-bound conformer (due to a high background level of magnesium and oscillations in calcium concentrations), we next monitored oxidation of Zn^2+^(Mg^2+^)-bound and Zn^2+^(Ca^2+^)-bound forms of the protein (Figure 4B), which can be generated from these conformers upon increased intracellular zinc concentrations (for details regarding NCS-1 conformers, see [12]). For all these forms, the dimer fraction did not exceed 30% of the total protein, whereas Zn^2+^(Mg^2+^)-bound NCS-1 was recognized as being most susceptible to disulfide dimerization, in accord with the results of our cell-based assays (see Figure 1B).

To assess the reduction stability of dNCS-1 in the presence of different metals, we next estimated redox potentials (E_h_) of the disulfide bond by monitoring equilibrium between dNCS-1/NCS-1 and glutathione redox pair GSSG/GSH, according to the method developed in our previous study [33]. Since probing of Zn^2+^-bound conformers was not possible due to the high affinity of zinc towards GSH [50], the experiments were performed only for Ca^2+^-bound and Mg^2+^-bound proteins (Figure 4C). In the case of Ca^2+^-bound NCS-1, a cooperative transition between dimeric and monomeric forms was observed at log([GSH]^2^/[GSSG]), ranging from −6 to −3, which corresponds to redox potentials from −100 mV to −220 mV. In the presence of Mg^2+^, dNCS-1 became completely reduced at E_h_ < −180 mV. These data indicate that under physiological redox conditions, both conformers can partially exist as dNCS-1, but the binding of calcium increases the lifespan of the dimer.

Since zinc is a redox-neutral metal, its binding can stimulate disulfide dimerization of NCS-1 only via structural effects, such as by enhancing surface accessibility of the SH group in the respective conformer or by promoting the formation of a non-covalent dimer, bringing together two SH groups. To test the first option, we probed various forms of NCS-1 using 5,5′-dithiobis-2-nitrobenzoic acid (DTNB, Ellman’s reagent), which differently modifies thiol groups in proteins, depending on steric factors [51,52]. Yet, none of the NCS-1 forms exhibited significant differences in reactivity with DTNB (Figure 4D). Given that the disulfide dimerization of NCS-1 relies on its structure (see Figure 4A), these data suggest that the generation of an intermolecular disulfide bond is preceded by the formation of a non-covalent dimer. Such dimerization is most favorable in the presence of Zn^2+^. Indeed, according to the results of analytical gel filtration of NCS-1 under non-oxidizing conditions, apo- and Zn^2+^-bound NCS-1 exist mainly as a non-covalent dimer, while the addition of Ca^2+^ prevents dimerization (Supplementary Figure S2).

The findings indicated that, overall, dNCS-1 is built via the formation of a non-covalent dimer, which is subsequently stabilized by a disulfide bond involving C38. Disulfide dimerization is more favorable in the presence of free Zn^2+^ (characteristic of oxidative stress), whereas binding of Ca^2+^ to NCS-1 inhibits this process. In contrast, the coordination of calcium in dNCS-1 increases its resistance to reduction and prolongs the lifespan of the dimer.

### 2.5. Calcium-Binding and Structural Properties of dNCS-1

**Figure 5:**
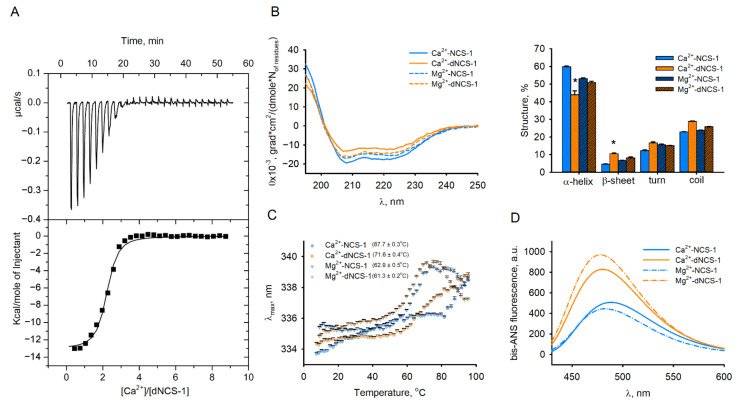
Calcium-binding and structural properties of dNCS-1 in vitro. (**A**) Binding of calcium to dNCS-1 was monitored using ITC. The top plot represent heat pulses and the bottom plot represents the binding isotherm along with the best fit curve, according to the “one set of sites” model. (**B**) Secondary structure elements of NCS-1 and dNCS-1 in the presence of 1 mM Mg^2+^ or 1 mM Ca^2+^, were determined from CD spectra (left) and presented as fractions of major secondary structure elements (right). * *p* < 0.05 as compared to NCS-1 parameters. (**C**) Thermal denaturation of NCS-1 and dNCS-1 was monitored in presence of 1 mM Mg^2+^ or 1 mM Ca^2+^ by intrinsic tryptophan fluorescence. The maximal wavelength of fluorescence emission (λ_max_) was plotted against temperature. Mid-transition temperatures calculated from the curves (±SE) are presented in the brackets. (**D**) Fluorescence spectra of complexes of bis-ANS with NCS-1 or dNCS-1 were recorded in the presence of 1 mM Mg^2+^ or 1 mM Ca^2+^.

To predict the effect of oxidation on the behavior of NCS-1 in cells, we next characterized metal ion-binding and structural properties of the disulfide dimer. For this purpose, dNCS-1 was prepared via mild oxidation of a recombinant, purified apo-form of NCS-1 during dialysis, followed by separation of the residual monomer by gel filtration. To correctly compare the properties of dNCS-1 and NCS-1, the amounts of these proteins taken in all subsequent experiments were normalized per monomer. According to isothermal titration calorimetry (ITC) data, dNCS-1 coordinated two calcium ions, i.e., one Ca^2+^ per monomer instead of three Ca^2+^ in the reduced protein (Figure 5A, Table 1) [12,13]. The K_D_ value of the single Ca^2+^-binding site of dNCS-1 was close to that of the high-affinity Ca^2+^-binding sites of the monomer (560 nM).

The general structural properties of dNCS-1 were characterized using a set of spectroscopic techniques. It was found that disulfide dimerization does not significantly affect the secondary structure and overall stability of Mg^2+^-bound NCS-1, as evidenced by CD spectroscopy and intrinsic fluorescence studies (Figure 5B,C). Meanwhile, it reduces the thermal stability of the Ca^2+^-bound conformer. Moreover, in the presence of calcium, dNCS-1 formation significantly altered the CD spectrum of the protein in the range of ~195–225 nm, i.e., at wavelengths where it is sensitive to the content of secondary structure elements [53]. The calculations using CDPro software (Colorado State University, Fort Collins, CO, USA) revealed that dNCS-1 formation results in ~27% decrease in the α-helical content and ~2.5-fold increase in the content of β-sheets. The latter effect suggested that dimerization might affect EF-hand motifs of the protein as these motifs represent the only sites containing such elements of secondary structure. Finally, according to experiments using hydrophobic fluorescent probe bis-ANS dye, *circa* 60–130% increase in its maximum fluorescence intensity upon the disulfide dimerization (Figure 5D) indicates that NCS-1 acquires enhanced surface hydrophobicity, which may have an impact on its affinity towards protein targets.

Summing up, disulfide dimerization reduces the stoichiometry of calcium binding to NCS-1 and results in the formation of a novel Ca^2+^-bound conformer of the protein, which might possess unique physiological activity.

### 2.6. Functional Properties of dNCS-1

**Figure 6:**
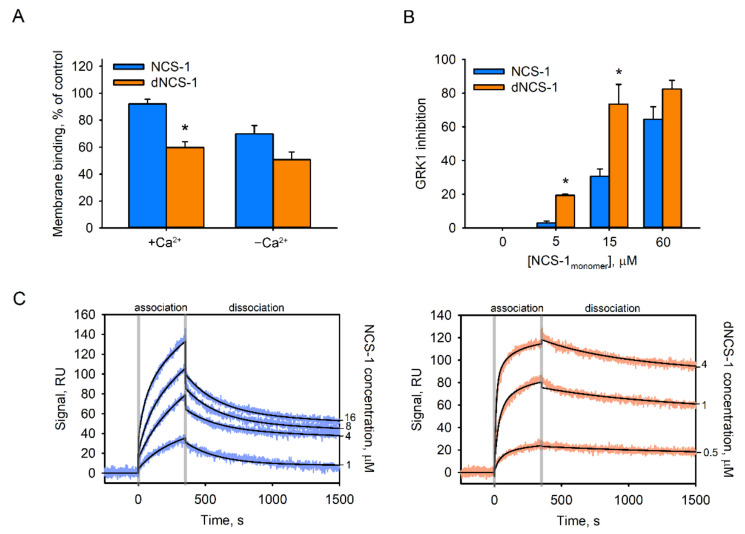
Functional properties of the NCS-1 disulfide dimer. (**A**) The binding of NCS-1 and dNCS-1 to urea-washed photoreceptor membranes was determined by equilibrium centrifugation assay in the presence of 20 mM MgCl_2_ and 1 mM CaCl_2_ (“+Ca^2+^”) of 1 mM EGTA (“−Ca^2+^”). The plots represent fractions of membrane-bound NCS-1 determined from at least three independent experiments. * *p* < 0.05 as compared to NCS-1 binding in the presence of Ca^2+^. (**B**) Inhibition of GRK-1 activity by Ca^2+^-bound forms of NCS-1 and dNCS-1 was examined in the reconstituted system containing urea-washed photoreceptor membranes (10 μM rhodopsin). * *p* < 0.05 as compared to effect of NCS-1. (**C**) Representative SPR sensorgrams demonstrate the interaction of Ca^2+^-bound NCS-1 (**left**) or dNCS-1 (**right**) with immobilized chimeric protein GRK1^N-C^.

The signaling function of NCS-1 is based on its three Ca^2+^-dependent activities, namely binding to cellular membranes (providing compartmentalization with a target), recognition of a target, and regulation of the activity of a target. To address alterations in these features of the protein, associated with its disulfide dimerization, we used functional assays based on components of the visual cascade, namely photoreceptor membranes/rhodopsin and rhodopsin kinase (GRK1). Indeed, NCS-1 displays a relatively high affinity towards photoreceptor membranes (comparable to those of other neuronal membranes), whereas GRK1 represents one of the structurally confirmed targets of the protein [10,54]. We found that disulfide dimerization produced a moderate negative effect on the membrane association of NCS-1 (Figure 6A). Unexpectedly, the dimer exhibited a prominent increase in Ca^2+^-dependent inhibitory activity against GRK1. In the presence of Ca^2+^-bound dNCS-1, rhodopsin phosphorylation was inhibited up to 10 times more effective than in the presence of monomeric protein (Figure 6B). This effect was related to the dramatically enhanced affinity of the inhibitory complex. According to the results of surface plasmon resonance (SPR) studies, the K_D_ of the complex of chimera GRK1^N-C^ (fusion protein containing regulatory domains of GRK1 [26]) with Ca^2+^-bound dNCS-1 had an order of magnitude higher than with the monomer (30.2 nM versus 590 nM) (Figure 6C, Table 2). Notably, SPR data obtained in the case of dNCS-1 could be reliably fitted using a “bivalent analyte” model, suggesting binding of two GRK1^N-C^ molecules per dimer. Overall, disulfide dimerization was found to markedly improve the inhibitory activity of NCS-1 against GRK1 due to a 20-fold higher affinity towards the enzyme.

### 2.7. Zinc-Binding Properties of dNCS-1

**Figure 7:**
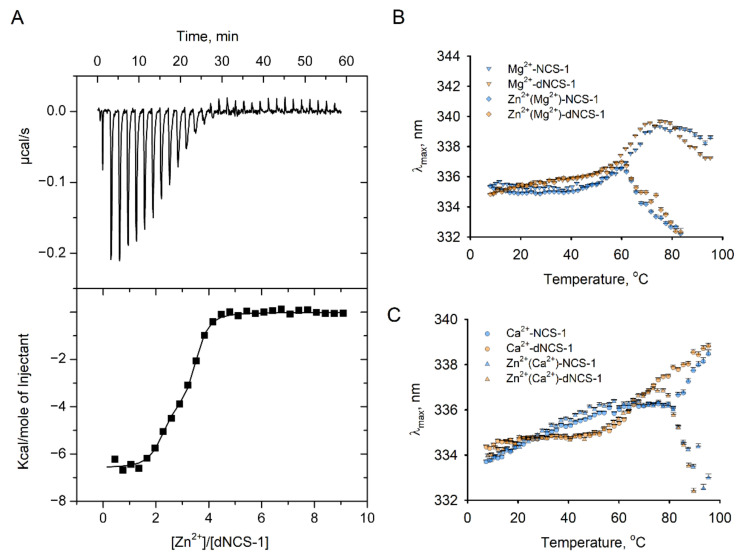
Zinc-dependent properties of dNCS-1 in vitro. (**A**) Binding of zinc to dNCS-1 was monitored using ITC. The top plot represents heat pulses and the bottom plot represents the binding isotherm along with the best fit curve, according to the “two sets of sites” model. (**B**,**C**) The impact of zinc on thermal denaturation and aggregation of NCS-1 and dNCS-1 loaded with Mg^2+^ (**B**) or Ca^2+^ (**C**) was monitored by intrinsic tryptophan fluorescence. The maximal wavelength of fluorescence emission (λ_max_) was plotted against temperature. The denaturation curves for Mg^2+^-bound and Ca^2+^-bound forms of NCS-1 and dNCS-1 (from Figure 5C) are provided for comparison.

As described above, disulfide dimerization of NCS-1 is markedly stimulated in the presence of zinc (see Figure 1 and Figure 4). Previously, we have found that monomeric NCS-1 is indeed capable of binding Zn^2+^ in high-affinity and low-affinity sites, with low-affinity binding promoting its aggregation (commonly observed for various neuronal proteins in neurodegenerative proteinopathies; see Section 3, Discussion) [12]. Our preliminary results indicate that dNCS-1 is accumulating in the retina of animals with a model of glaucoma (unpublished data), a neurodegenerative disorder associated with increased retinal concentrations of mobile zinc [42]. Considering these observations, we performed additional ITC experiments aimed at examining the Zn^2+^-binding properties of dNCS-1. Although monomeric protein was found to coordinate three zinc ions [12] (Table 3), dNCS-1 was incapable of filling all six potential sites. Instead, it bound two Zn^2+^ with a K_D_ of 12 nM, and one zinc ion with a K_D_ of 470 nM (Figure 7A, Table 3).

Given the destabilizing and pro-aggregative effects of the low-affinity Zn^2+^ binding to NCS-1 [12], we also assessed how saturation with zinc of physiologically relevant Mg^2+^-loaded (Figure 7B) and Ca^2+^-loaded (Figure 7C) forms of dNCS-1 would affect their thermal stability. Monitoring of the thermal dependence of tryptophan fluorescence revealed that in both cases, zinc induced aggregation of dNCS-1, as evidenced by a sharp decrease in λ_max_ values at high temperatures. A similar effect was observed for monomeric NCS-1, in agreement with previous findings [12]. For both NCS-1 forms, Ca^2+^-loaded conformers were more resistant to zinc-induced aggregation than Mg^2+^-loaded forms. Meanwhile, the aggregation of Zn^2+^(Ca^2+^)-dNCS-1 started at a lower temperature (75 °C) than in the case of Zn^2+^(Ca^2+^)-NCS-1 (83 °C).

Thus, dNCS-1 is capable of binding zinc via a different mode of interaction, as compared to NCS-1. Synergetic effects of oxidation, calcium-binding, and subsequent zinc-binding increase susceptibility of NCS-1 to aggregation.

### 2.8. Modeling of dNCS-1 Structure

**Figure 8:**
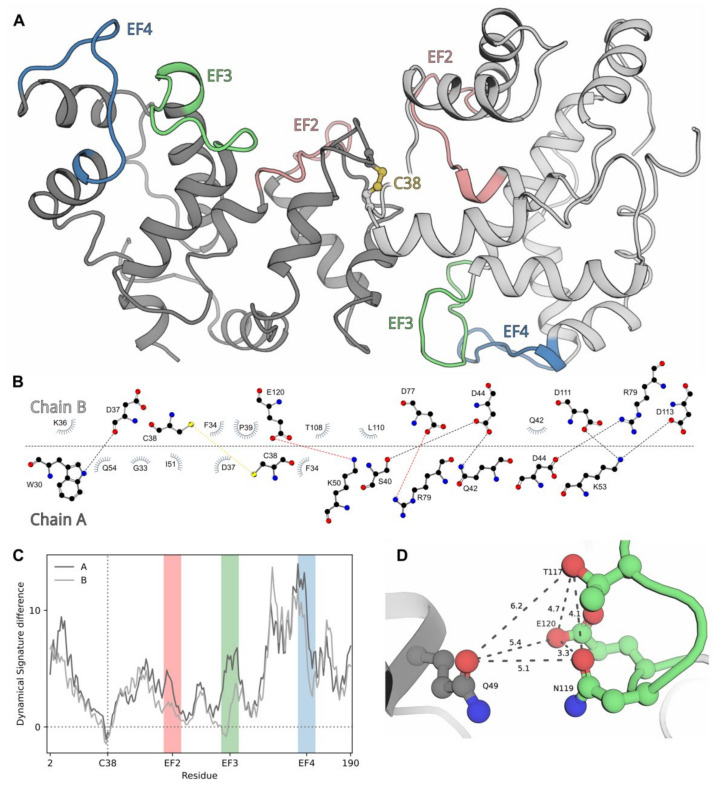
Structural organization of dNCS-1 as predicted by molecular modeling. (**A**) The tertiary structure of dNCS-1. The covalent bond between C38 of both chains is shown in yellow. EF2, EF3, and EF4 are colored in red, green, and blue, correspondingly. (**B**) Schematic representation of the contacts forming an intermonomer interface. Hydrogen bonds, salt bridges, and the disulfide bridge are colored in gray, red, and yellow, correspondingly. Hydrophobic contacts are depicted with gray combs. (**C**) Analysis of Dynamical Signatures (e.g., flexibility) was performed for individual subunits of dNCS-1 in comparison with free monomers. (**D**) Structure or the potential Zn^2+^-binding site localized at the intermonomer interface. Distances between the atoms are shown in angstroms.

To assess structural aspects underlying the unique properties of dNCS-1, we performed molecular modeling of disulfide dimer of human NCS-1, based on NMR structures of Ca^2+^-free myristoylated NCS-1 from *Schizosaccharomyces pombe* (PDB 2L2E). The Ca^2+^-free conformer was chosen for the following reasons. Firstly, dNCS-1 can be generated in solution in the absence of calcium (see Figure 4). Secondly, dNCS-1 binds one Ca^2+^ per monomer (see Table 1) and is unlikely to adopt the conformation peculiar to reduced Ca^2+^-loaded NCS-1, containing three calcium ions (PDB 2LCP, 4GUK, 4YRU, 5AEQ). Thirdly, dimerization of Ca^2+^-free NCS-1 may constitute an intermonomer Zn^2+^-binding site, the filling of which may facilitate the formation of dNCS-1. Rigid-body docking of two molecules of human Ca^2+^-free NCS-1, built based on the yeast protein, was performed using two different algorithms (ZDOCK and HADDOCK) and independently yielded an asymmetric dimer of NCS-1 (Figure 8A). The best structures (with cysteines on the dimer interface and minimal distance between their S atoms), generated in each case, were quite similar, with small differences only in the relative orientation of the subunits due to the rigid-body docking constraints. The most relevant dimer contained an intermonomer interface formed by a number of hydrophobic and polar contacts, involving three key residues from the coordination sphere of EF3 (D111, D113, and E120) in one of the subunits (chain B, Figure 8B). Analysis of the dynamical signatures of the individual NCS-1 chains within the dimer, in comparison to free monomer, revealed a region with expectedly increased rigidity around C38 residues, forming a covalent bond (Figure 8C). At the same time, the other parts of both subunits exhibited elevated mobility, especially in N-terminal and C-terminal parts. Each subunit demonstrated a unique pattern of mobility, in agreement with the asymmetric structure of the dimer. In summary, the results of molecular modeling suggest that NCS-1 forms an asymmetric dimer with increased conformational flexibility of the subunits.

### 2.9. Role of NCS-1 Disulfides in Oxidative Stress-Induced Apoptosis

**Figure 9:**
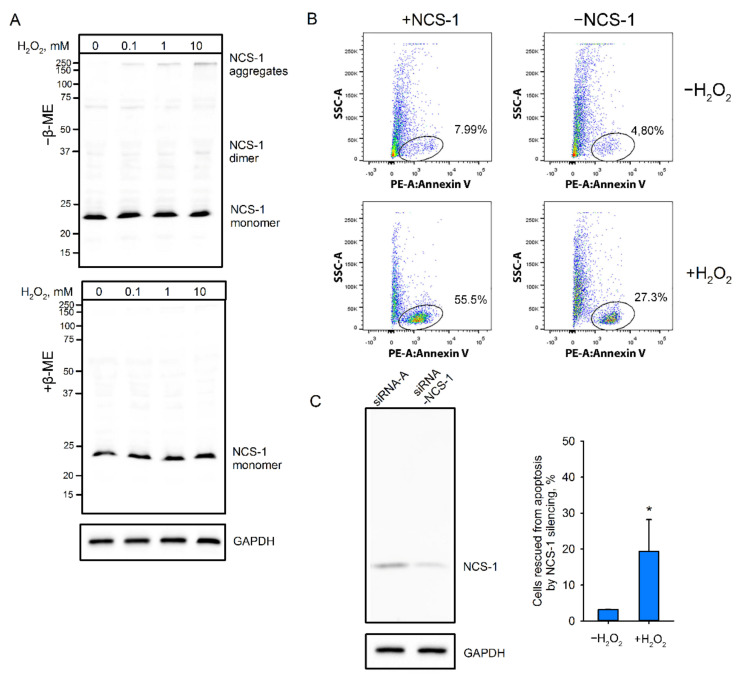
Effect of NCS-1 silencing on oxidative stress-induced apoptosis in Y79 retinoblastoma cells. (**A**) The cells were incubated in the presence of increasing concentrations of H_2_O_2_ for 30 min. Disulfide forms of NCS-1 were detected by Western blotting of cell lysates conducted under non-reducing (“−β-ME”) or reducing (“+β-ME”) conditions (**top**). (**B**) The cells were incubated under normal or oxidizing (1 mM H_2_O_2_) conditions with (−NCS-1) or without (+NCS-1) NCS-1 silencing. The population of apoptotic cells was assessed by flow cytometry, using fluorescently labeled annexin V. (**C**) NCS-1 expression in the presence of non-specific (lane “siRNA-A”) or NCS-1-specific (lane “NCS-1”) si-RNA was examined by Western blotting (**left**). Effect of NCS-1 silencing on cell survival under normal (“−H_2_O_2_”) and oxidative (“+H_2_O_2_”) conditions (**right**). * *p* < 0.05.

As described above, extensive oxidation of NCS-1 in cells results in the conversion of the protein into disulfide-dependent aggregates, which are utilized by the proteasome (see Figure 2C). Since proteasome overload with oxidized proteins represents one of the signals triggering apoptosis [55,56,57], we hypothesized that excessive accumulation of NCS-1 disulfides may contribute to this mechanism. To test this hypothesis, we used a model based on Y79 retinoblastoma cells showing prominent endogenous expression of NCS-1 [58]. Being in oxidative stress, the Y79 cells exhibited accumulation of dNCS-1 as well as other disulfide-dependent forms of the protein, mainly high-molecular-weight aggregates (Figure 9A). In this respect, Y79 differed slightly from HEK293 (probably due to differences in redox homeostasis in cancer cells), suggesting that, in the first case, the proteasome system was not able to fully utilize NCS-1-containing disulfide aggregates. Consistently, oxidative conditions significantly increased the amount of Y79 cells undergoing apoptosis, as revealed by flow cytometry (Figure 9B). Interestingly, NCS-1 silencing using specific RNA interference (see Figure 9C) reduced the susceptibility of Y79 cells to apoptosis. Downregulation of NCS-1 expression by 2.5 times was found to decrease the number of apoptotic cells twofold. This effect was most pronounced under conditions of oxidative stress. The fraction of Y79 cells rescued from hydrogen peroxide-induced apoptosis by NCS-1 knockdown increased from approximately 3% to 20% of the total cellular population. Based on these observations, we propose the existence of an NCS-1-dependent apoptosis-triggering mechanism, which may involve the formation of disulfide forms of the protein.

## 3. Discussion

This study is the first to discover that ubiquitous neuronal Ca^2+^-sensor protein NCS-1 possesses redox sensitivity in response to oxidizing conditions by the formation of disulfide dimers with altered structural properties and functional activity. In this respect, NCS-1 resembles some members of the NCS family (recoverin and VILIP-1) and several other EF-hand proteins, such as S100 proteins, oncomodulin, secretagogin, and others [27,28,30,31,32,33,59,60,61]. Disulfide bonds in these proteins are commonly formed by conserved cysteine residues localized in their non-functional EF-hands. Consistently, it has been suggested that in the course of evolution, they sacrificed the ability to bind calcium to acquire redox sensitivity [60]. However, such a tendency is not always observed, even within the NCS family: NCS-1 and recoverin form disulfide dimers via their single conservative cysteine in EF1, whereas VILIP-1 uses its unique C-terminal cysteine [28,30,31]. Consistently, the non-covalent dimeric forms of NCS proteins, the formation of which precedes disulfide bonding, significantly differ in the overall structural organization [62].

Despite structural differences, the formation of disulfide dimers of NCS-1 and other NCSs seems to follow a similar pattern, namely non-covalent complexing of two protein molecules and subsequent intermolecular disulfide bonding [28,32]. Generally, SH groups react with intracellular two-electron oxidants (such as hydrogen peroxide) to form sulfenic acid, which (in the presence of the second juxtaposed SH group) can form a disulfide bond or otherwise oxidize irreversibly, yielding sulfinic or sulfonic acids [63]. Therefore, the generation of disulfide dimers is governed by two factors, namely affinity of the non-covalent complex, which brings together two thiol groups, and their reactivity, which depends on pKa and other properties. In NCSs, the first factor seems to take precedence over the second one. For instance, in the case of recoverin, oxidation proceeds more efficiently for a Ca^2+^-free conformer, whereas formation of the dimer is more favorable in Ca^2+^-bound protein [28,64]. In NCS-1, we did not observe any preference in terms of the reactivity of the thiol group among the Ca^2+^-bound, Mg^2+^-bound, or Zn^2+^-bound conformers. Meanwhile, Zn^2+^-bound forms seemed to be most susceptible to disulfide dimerization. This phenomenon can be partially explained assuming the existence of a potential Zn^2+^-binding site localized at the intermonomer interface. Indeed, close examination of the EF3 region of chain B (the second region exhibiting increased rigidity) revealed an environment similar to a cation-binding site (Figure 8D), in particular, the Zn^2+^-binding site of the E1Q1 family [65]. Filling of this site (manifested in ITC studies as the site with a stoichiometry of single ion per dimer, K_D_ = 4.7 × 10^−7^) can enhance non-covalent dimerization of NCS-1, thereby promoting the formation of the disulfide bond. Similar sites were found, for instance, in EF-hand proteins of the S100 family [66]. The described effect of zinc could be observed only in the case of Ca^2+^-free NCS-1 since the presence of calcium inhibits disulfide dimerization. Accordingly, Ca^2+^ binding was shown to prevent the formation of a non-covalent dimer of NCS-1 due to steric restrictions provided by its C-terminal segment [54]. It should be added that according to our previous study, each NCS-1 molecule contains three Zn^2+^-binding sites in EF2-EF4, suggesting six potential sites in dNCS-1 [12]. Assuming that dNCS-1 is asymmetric (see Figure 8), whether these sites and/or potential intermonomer sites will be occupied by three zinc ions remains an open question. Yet, since metal coordination in EF3 and EF4 of dNCS-1 might be hindered (see below), we speculate that two zinc ions bind to EF2 of each subunit, whereas the remaining zinc ion can be coordinated in the intermonomer site.

The coordination of calcium in dNCS-1 stabilized this form, highlighting structural differences between Ca^2+^-loaded monomeric (three Ca^2+^ per molecule) and disulfide dimeric (one Ca^2+^ per subunit) protein. The reduced stoichiometry of Ca^2+^ binding in dNCS-1 could be associated with the fact that the dimer interface involved three key residues (D111, D113, and E120) from the coordination sphere of EF3 (Figure 8B), which disabled this site. The second disabled site was unlikely to have been EF2 since dNCS-1 retained the ability to bind GRK1, which would be difficult in the absence of calcium in EF2. In contrast, the calcium affinity of EF4 was more likely to have been diminished due to the dramatically increased flexibility of the respective region in dNCS-1 (Figure 8C). Despite the decreased stoichiometry of calcium-binding, the total Ca^2+^ sensitivity of dNCS-1 seemed to increase as the dimer lost low-affinity Ca^2+^-binding sites while retaining regulatory activity.

Another phenomenon seen in this study is the dramatically enhanced affinity and inhibitory activity of dNCS-1 against GRK1. Generally, residues of the dNCS-1 interface did not interfere with the GRK1-binding site [54], as far as could be predicted in the absence of a Ca^2+^-bound dimer structure. In any case, the increased structural plasticity suggested for dNCS-1 could have enhanced the stability of its complex with GRK1 and could be part of the conformational selection process [67,68], which seems relevant for the interactions of multifunctional NCS-1. An additional factor could be seen from the SPR studies, revealing 10-fold faster kinetics of GRK1 in association with dNCS-1, as compared to NCS-1 (see Table 2). This effect may be related to the acceleration of binding of the second subunit of the dimer upon capturing the first subunit on the GRK1-coated chip. A similar effect may occur with respect to GRK1 activity, as rhodopsin represents a dimer [69], and dNCS-1 can simultaneously inhibit two molecules of the receptor-bound activated enzyme.

What are the conditions favoring the formation and accumulation of dNCS-1 in living cells? As noted above, Ca^2+^-bound dNCS-1 is more resistant to reduction than its Mg^2+^-bound form. Nevertheless, under redox conditions corresponding to non-dividing (differentiated) cells (E_h_GSSG/GSH ~ −220 mV [70]), even in the presence of Ca^2+^, dNCS-1 constituted merely 5–10% of the total NCS-1 (see Figure 4C). In proliferating cells (baseline E_h_GSSG/GSH ~ −260 mV [70]), such as HEK293, a similar amount of dNCS-1 was formed only upon exposure to hydrogen peroxide (see Figure 1A). The situation changed in the presence of zinc, which did not affect the reactivity of C38 of NCS-1 while increasing the affinity of its non-covalent dimer. Indeed, in this case, the fraction of dNCS-1 in HEK293 cells incubated with hydrogen peroxide could reach approximately 30% (Supplementary Figure S1), indicating that Zn^2+^ binding results in the decreased redox potential of the NCS-1/dNCS-1 pair. In the absence of zinc, similar dimer content was observed at E_h_GSSG/GSH > −180 mV (see Figure 4C), which approached values characteristic of apoptosis (from −150 to −170 mV) [70,71,72]. However, at high free zinc concentrations, disulfide dimerization of NCS-1 occurs under relatively mild oxidative conditions and can possibly participate in redox regulation mechanisms. Normally, given the high cytotoxicity of free zinc, it is buffered by specialized proteins and low-molecular-weight chelators [44,50]. Yet, for instance, in gluzinergic neurons of the brain or retina, Zn^2+^ acts as a neurotransmitter and can enter the cytoplasm of post-synaptic cells through the ionotropic glutamate receptors. This effect increases the cytoplasmic pool of so-called loosely-bound zinc, which can interact with different signaling proteins like NCS-1 [40,73]. Thus, the conditions favoring dNCS-1 formation can be created in living neurons. This further supports the argument that the dimer may play a role in the mechanisms of redox regulation and is in line with our observation that the disulfide dimerization of NCS-1 is actually a reversible process, occurring with the participation of the thioredoxin system, a common component of redox regulation [74].

While the accumulation rate of dNCS-1 in living cells is relatively low, it displays increased Ca^2+^ sensitivity and regulatory activity, as compared to the reduced NCS-1. What might be the specific physiological function of this enhanced form of the protein? Previous studies using cellular and animal models have shown that in neurons and cardiomyocytes, NCS-1 exhibits protective activity against various types of stress [35,36,75]. Similar activity on the part of this protein has been demonstrated in breast cancer cells [76]. These observations generally agree with our data obtained using the Y79 model, in that NCS-1 is involved in signaling pathways regulating cell death/survival decisions. Most commonly, the cytoprotective effects of NCS-1 are explained by its participation in the PI3K/Akt signaling pathway [35,36,76,77]. Indeed, in the presence of calcium, NCS-1 upregulates the content of PI(3,4,5)P_3_ in the plasma membrane via direct activation of PI4K [78], and this effect might induce membrane recruitment and phosphorylation/activation of Akt. For instance, overexpression of NCS-1 in neurons increases the level of phospho-Akt, simultaneously accelerating axonal growth and restoring cellular function after an injury [77]. It is apparent that to efficiently utilize the protective function of NCS-1, the cells increase the expression of this protein in response to stress conditions [35,36,79]. We would argue that enhanced activity of dNCS-1, which is generated under oxidizing conditions, represents a similar feedback mechanism, facilitating the function of the already available protein.

Although, in this study, hyperactivity of dNCS-1 was demonstrated only in relation to GRK1, one may hypothesize such an effect in respect of other Ca^2+^-dependent targets of the protein, at least due to increased Ca^2+^ sensitivity of the dimer. Previous works have reported that Ca^2+^-loaded NCS-1 can directly bind not only GRK1 but also GRK2, and this binding attenuates phosphorylation (desensitization) of its substrate GPCRs [8,54,80,81,82]. If dNCS-1 were to exhibit excessive regulation of GRK2 as it does in respect of GRK1, this might significantly reflect on the regulation of stress responses. Indeed, overexpression of GRK2 in cardiomyocytes and microglial cells leads to an increase in reactive oxygen species (ROS) production and apoptosis, which can be prevented by GRK2 silencing or inhibition [83,84]. Furthermore, GRK2 directly interacts with Akt, and this interaction inhibits Akt activity [85]. In this case, it is the increased inhibitory activity of dNCS-1 against GRK2 that may directly mediate the cytoprotective activity of the protein.

Interestingly, stimulation of the PI3K/Akt pathway can also activate GSK3β, which, in contrast, mediates neuronal death in Alzheimer’s disease and Parkinson’s disease [86,87]. The protective activity of dNCS-1 can actually be reversed under certain conditions, especially upon generation and accumulation of various disulfide forms of the protein. Indeed, the formation of intermolecular disulfide bonds is a frequent phenomenon observed in neurodegenerative proteinopathies, neurological disorders associated with aggregation, and/or misfolding of intracellular and/or extracellular proteins. These proteins include the following: SOD and TDP-43 in ALS; amyloid-beta, tau, and p75 neurotrophin receptors in Alzheimer’s disease; synuclein in Parkinson’s disease; HTT in Huntington’s disease; androgen receptors in spinal and bulbar muscular atrophy; prion in prion-related diseases; ataxins in spinocerebellar ataxias [88,89], myocilin in glaucoma [90], and apparently recoverin and arrestin in AMD [33,91]. Interestingly, many proteinopathies are associated with aberrantly increased levels of free zinc, whereas respective disulfide-forming proteins commonly bind this metal, thereby mediating its neurotoxic effects [40,42,45,92,93,94]. The joint action of two factors, namely excessive Zn^2+^ binding and disulfide bonding, increases the susceptibility of certain neuronal proteins to misfolding and aggregation, the well-established hallmarks of neurodegeneration. Our current data indicate that NCS-1 may be sensitive to both of these factors (see Figure 2C, Figure 3, Figure 7B and Figure 9A), which would make it one of the proteins involved in mechanisms of neurodegeneration.

Indeed, application of the Y79 model indicates that Zn^2+^ binding and subsequent disulfide bonding of NCS-1 may promote oxidative stress-induced cell death, as knockdown of this protein rescues cells from apoptosis under oxidizing conditions. At first glance, this finding contradicts previous observations suggesting the general cytoprotective nature of NCS-1 (see above). However, this protective activity might only be characteristic of the reduced protein or, alternatively, may be associated with the generation of the hyperactive dNCS-1 during initial cellular responses to oxidative stress. Meanwhile, in developed phases of this stress, characterized by both increased redox potential and zinc concentrations, accumulation of multiple disulfide forms of NCS-1 (including its aggregates) may instead produce pathological effects. Such behavior is indeed characteristic of different neuronal proteins, including NCS-1 homologs. For instance, VILIP-1, initially forming disulfide dimers with altered target (guanylyl cyclase B) regulation properties, can also accumulate in the aggregated form found in the spinal cord of ALS patients [30,31]. Similarly, mild oxidation of recoverin produces its disulfide dimer with altered activity, whereas extensive light illumination of the retina (yielding oxidative conditions characteristic of AMD) induces accumulation of disulfide aggregates of the protein [27,33]. Interestingly, in the latter case, a significant portion of recoverin binds to the proteasome, forming disulfide bonds with its regulatory subunits [33]. Meanwhile, proteasome overload, with oxidized and/or aggregating proteins (such as NCS-1 homolog GCAP2, TDP-43, tau, and others) and an unfolded protein response (UPR), is known to trigger signaling mechanisms, leading to apoptosis [55,56,57].

According to our data, in HEK293 cells, dNCS-1 is degraded by the proteasome, while apoptosis is the outcome that accompanies dNCS-1 formation in Y79. Retinoblastoma cells express not only NCS-1 but also recoverin, both of which form disulfide aggregates and may collectively affect proteasome capacity [58,95]. Notably, NCS-1 oxidation in Y79 cells is not limited to disulfide dimerization, as in HEK293 cells, but is accompanied by the formation of disulfide aggregates and other disulfide forms. This phenomenon can be explained by the well-known fact that malignant cells, such as Y79, possess imbalanced redox homeostasis, which may lead to increased protein oxidation, especially after an additional hydrogen peroxide stimulus [96]. The forming NCS-1 aggregates can be targeted at the proteasome, resulting in its overload, UPR, and apoptosis. In HEK293, we only observed a similar pattern when proteasomes were inhibited. Yet, even in this case, we detected an accumulation of dense NCS-1-positive bodies in the perinuclear zone, which can represent autophagosomes or NCS-1-containing aggresomes, insoluble deposits often found in neurons in Parkinson’s disease, Alzheimer’s disease, ALS, or retinitis pigmentosa [97,98,99]. A key protein, linking the proteasome system and neurodegeneration, is the ubiquitin ligase Parkin, which targets different proteins for degradation, while its mutant forms are associated with Parkinson’s disease [57]. Its function includes modulation of mitophagy, the autophagy pathway responsible for the utilization of damaged mitochondria [100]. Parkin is phosphorylated by protein kinase PINK1, which thereby regulates its ubiquitin ligase activity and mitophagy [101]. Notably, PINK1 is a well-recognized target of NCS-1 [102]. Thus, hypothetical dysregulation of PINK1 by oxidized NCS-1 may disturb Parkin-dependent protein quality control and mitophagy, thereby aggravating the apoptosis triggered by proteasome overload and UPR.

The abnormal effect of dNCS-1 on GRKs, in this case, may contribute to accompanying mechanisms. The observed pro-apoptotic action of the protein is unlikely to be associated with its abnormal activity in respect of GRK1, which seems to be absent in Y79 cells. However, these cells express other members of the GRK family, including GRK2, GRK3, and GRK6 [58,95]. Possible excessive inhibition of GRK2 by dNCS-1 may, for instance, attenuate desensitization of the CRF1 receptor, thereby hindering the adaptation of Y79 cells to stress conditions [103,104]. Previous studies have consistently proposed suppression of CRF1 receptor signaling as an approach to combating oxidative stress in Alzheimer’s disease [105].

In summary, our observations provide a rationale for the following conclusions. NCS-1 is a redox-regulatory protein, which responds to oxidizing conditions and Zn^2+^ binding by the formation of structurally determined dNCS-1 (a unique form of the protein, exhibiting an increased sensitivity to calcium signals and regulatory activity against GRK1), as well as other potential NCS-1 targets. Under normal conditions, these properties can enhance the cytoprotective function of NCS-1, whereas an excess of the dimeric form can be recycled by the thioredoxin system. With more pronounced oxidative stress and an abundance of loosely-bound zinc, accumulating dNCS-1 can form cytotoxic disulfide-dependent aggregates, overloading proteasomes and triggering apoptosis. Further studies are required to confirm the revealed redox-dependent properties of NCS-1 and verify the proposed mechanisms in animal models.

## 4. Materials and Methods

### 4.1. Materials

All reagents and kits for plasmid construction were from Evrogen (Moscow, Russia) or Promega (Madison, WI, USA). Chromatography resins and pre-packed columns were from GE Lifesciences (Chicago, IL, USA). Reagents and consumables for Western blotting and surface plasmon resonance spectroscopy were from Bio-Rad (Hercules, CA, USA). Culture media and reagents for cellular biology were from Gibco (Amarillo, TX, USA) and PAN Biotech (Aidenbach, Germany). Secondary antibodies conjugated with peroxidase were from Jackson Immunoresearch (West Grove, PA, USA). TurboFect transfection reagent, bicinchoninic acid (BCA) Protein Assay Kit and Recombinant GRK1, goat anti-rabbit Alexa Fluor 555-conjugated IgG, and FITC-conjugated phalloidin were from Thermo Fisher (Waltham, MA, USA). Reagents for NCS-1 RNA silencing were from Santa Cruz Biotechnology (Dallas, TX, USA). Equipment and reagents for microscopy were from Zeiss (Oberkochen, Germany). Reagents for flow cytometry and DAPI were from BD Biosciences (Franklin Lakes, NJ, USA). [γ^32^P]-adenosine triphosphate was provided by Shemyakin-Ovchinnikov Institute of Bioorganic Chemistry RAS (Russia). Ionomycin, auranofin, EDTA-free protease inhibitor cocktail, and Ellmann’s reagent (5,5′-dithiobis(2-nitrobenzoic) acid) were from Sigma-Aldrich (St. Louis, MO, USA). Iodoacetamide was from MP Biomedicals (Santa Ana, CA, USA). Other chemicals were from Sigma-Aldrich, AppliChem (Darmstadt, Germany), or Amresco (Dallas, TX, USA) and were at least of reagent grade.

### 4.2. Generation of Plasmid Constructs

Plasmid pET22b for bacterial expression of NCS-1 was previously constructed in our laboratory [8]. To obtain a vector for NCS-1 expression in HEK293 cells, its gene was then subcloned into the pCI-neo vector between NheI and XbaI restriction sites. The resulting construction was amplified in *Escherichia coli* cells (strain XL1 blue) and purified from a bacterial culture with a Plasmid Miniprep kit. pET28a plasmid encoding GRK11-191,512-557 (GRK1N-C) fusion protein was generated based on full-length GRK1 gene, as described in [26].

### 4.3. Bacterial Expression and Purification of Recombinant Proteins

Expression and purification of recombinant NCS-1 were performed according to a recently proposed procedure, yielding protein product with a high degree of N-terminal myristoylation (~98%) [106]. Fusion protein GRK1^N-C^ with C-terminal His-tag was expressed in *E. coli* and purified from bacterial lysate under non-denaturating conditions as described in [26]. Protein concentrations were determined using BCA Protein Assay Kit (Thermo Fisher, Waltham, MA, USA) or spectrophotometrically at 280 nm using extinction coefficients of 21,430 M^−1^ cm^−1^ for NCS-1 and 33,640 M^−1^ cm^−1^ for GRK1^N-C^ [26,107].

### 4.4. Oxidation of NCS-1 In Vitro

Reduced NCS-1 (20 μM) was incubated with 0.1–1000 μM H_2_O_2_ in 20 mM Tris-HCl buffer (pH 8.0), 150 mM NaCl, containing one of the following components: 1 mM MgCl_2_; 1 mM CaCl_2_; 1 mM MgCl_2_ and 0.1 mM ZnCl_2_; 1 mM CaCl_2_ and 0.1 mM ZnCl_2_; 0.1 mM ZnCl_2_. For the control samples, 1% SDS was added to the reaction mixture. Samples were incubated in a thermostatic shaker (37 °C, 1000 rpm) for 30 min, diluted with 1 volume of sample buffer without β-mercaptoethanol, and analyzed with SDS-PAGE. Dimer weight fractions were quantified with GelAnalyzer.2010a software (Istvan Lazar Jr., University of Budapest, Budapest, Hungary) for densitometric analysis (http://www.gelanalyzer.com/, accessed on 2 December 2019).

### 4.5. Ellmann’s Assay

Accessibility of the C38 sulfhydryl group of NCS-1 was assessed by colorimetric assay involving Ellmann’s reagent (5,5′-dithiobis(2-nitrobenzoic) acid). The reagent (final concentration 0.5 mM) was added to reaction mixtures containing 20 μM NCS-1 in 20 mM Tris-HCl buffer (pH 8.0), 150 mM NaCl, containing one of the following components: 1 mM MgCl_2_; 1 mM CaCl_2_; 1 mM MgCl_2_ and 0.1 mM ZnCl_2_; 1 mM CaCl_2_ and 0.1 mM ZnCl_2_; 0.1 mM ZnCl_2_. The reaction was monitored for 3 min by measuring the absorption at 412 nm. The kinetic curves were plotted based on 3 independent measurements, using the SigmaPlot 12.5 software (Systat Software, Chicago, IL, USA).

### 4.6. Preparation of dNCS-1

Purified recombinant NCS-1 (2 mg/mL) was dialyzed against 20 mM Tris-HCl buffer (pH 8.0), containing 150 mM NaCl, 1 mM H_2_O_2_ at 4 °C, and vigorous stirring for 3 h. The resulting dialysis mixture contained about 80% of dNCS-1 as determined by SDS-PAGE under non-reducing conditions. To remove residual hydrogen peroxide, the sample was dialyzed against 20 mM Tris-HCl buffer (pH 8.0), two times for 3 h, aliquoted, and stored at –80 °C. In some cases, dimer was separated from residual monomer by gel filtration on Superdex 75 10/300 GL column, equilibrated with 20 mM Tris-HCl buffer (pH 8.0), 150 mM NaCl, 0.5 mM EDTA, at 0.5 mL/min flow rate. The same procedure was utilized for analytical chromatography of non-covalent dimers of NCS-1 performed in 20 mM Tris-HCl-buffer (pH 8.0), 150 mM NaCl, 1 mM DTT, in presence of calcium (0.1 mM CaCl_2_), zinc (0.1 mM ZnCl_2_), or 1 mM EGTA.

### 4.7. Evaluation of Redox Potential of dNCS-1

Redox equilibrium between Ca^2+^/Mg^2+^-loaded forms of dNCS-1/NCS-1 and GSSG/2GSH redox pair was monitored using the previously described procedure [33]. Briefly, dNCS-1 (1.5 µM) in 50 mM HEPES buffer (pH 7.4), containing 100 mM KCl, and either 0.1 mM CaCl_2_ or 1 mM MgCl_2_, was mixed with 0.1 mM GSSG and various concentrations of GSH (8.8 µM–72.4 mM) under anaerobic conditions. The dissolved oxygen was removed from the solutions using a degassing station (TA Instruments, New Castle, DE, USA) at a pressure of 25 mm Hg for 20 min, under stirring at 500 rpm. The water stock used for the solutions was saturated with argon gas by bubbling 99.993% argon through the solution for 7–10 min. The oxygen-freed solutions were isolated from the air using sealing with argon. The reaction solutions sealed with argon were incubated at 35 °C for 62 h and subjected to SDS-PAGE under non-reducing conditions. The weight fractions of NCS-1 forms were evaluated densitometrically and analyzed in SigmaPlot 12.5 (Systat Software, Chicago, IL, USA). The redox potential of dNCS-1 was calculated exactly as described in a previous study for dimeric recoverin [33].

### 4.8. Fluorescence Measurements

The experiments were conducted in 10 mM HEPES buffer (pH 7.6), 150 mM KCl, containing MgCl_2_ (1 mM), CaCl_2_ (1 mM), or ZnCl_2_ (0.1 mM), or their combinations. Fluorescence emission spectra were recorded with a Cary Eclipse spectrofluorimeter (Varian Inc., Palo Alto, CA, USA), equipped with a Peltier-controlled cell holder, as previously described [8,12]. Tryptophan fluorescence of NCS-1 (14 μM) or dNCS-1 (7 μM) was excited at 280 nm, the acquired spectra (see Supplementary Figure S3) were fitted to log-normal curves using LogNormal software (IBI RAS, Pushchino, Russia) and the fluorescence spectrum maximum positions (λ_max_) obtained from these fits were plotted against temperature. The standard deviations of λ_max_ values did not exceed 0.41 nm. The mid-transition temperatures were calculated from the temperature dependence of λ_max_ using Boltzmann sigmoid with OriginPro 8.0 software (OriginLab, Northampton, MA, USA). Fluorescence spectra of surface hydrophobicity probe 4,4′-dianilino-1,1′-binaphthyl-5,5′-disulfonic acid (bis-ANS) were measured in samples containing 1 μM bis-ANS and 6 μM NCS-1 or 3 μM dNCS-1. bis-ANS fluorescence was excited at 385 nm. The concentration of bis-ANS in stock solution was determined using an extinction coefficient of 16,790 M^−1^ cm^−1^ [108]. The standard errors for estimates of maximum fluorescence intensity are within 1%.

### 4.9. Circular Dichroism (CD) Measurements

CD measurements were carried out with a JASCO J-810 spectropolarimeter (JASCO Inc., Tokyo, Japan) with a Peltier-controlled cell holder [29]. Samples contained NCS-1 (8 µM) or dNCS-1 (4 μM) in 10 mM HEPES (pH 7.6), 100 mM KCl with one of the following components: 1 mM MgCl_2_; 1 mM CaCl_2_; 1 mM MgCl_2_ and 0.1 mM ZnCl_2_; 1 mM CaCl_2_ and 0.1 mM ZnCl_2_. Buffer contribution was subtracted from the resulting spectra. Secondary structure fractions were assessed using the CDPro software package (Colorado State University, Fort Collins, CO, USA) [109].

### 4.10. Isothermal Titration Calorimetry (ITC)

The binding of metal ions to dNCS-1 was analyzed by ITC using the MicroCal iTC200 instrument (Malvern Pananalytical, Malvern, UK) as described previously [12]. Experiments were performed at 25 °C in 20 mM Tris-HCl buffer (pH 8.0), 150 mM NaCl. dNCS-1 (12.5 μM) in the calorimetric cell was titrated with injections of 750 μM CaCl_2_ or ZnCl_2_ (direct titration). Each titration peak was integrated and plotted as a function of the metal ion/dNCS-1 molar ratio. The baseline was plotted by injecting CaCl_2_ or ZnCl_2_ into the working buffer. Dissociation constants (K_D_), enthalpy changes (ΔH), and the number of binding sites (N) were determined by fitting the plots with “one set of sites” or “two sets of sites” models, using a non-linear least-squares minimization method (the best fit was determined from the lowest Chi^2 value). Stoichiometry of the metal binding was estimated based on N values calculated from direct titration experiments by considering dNCS-1 as a single molecule. Data analysis was performed in Origin 7.0 software with the ITC package (OriginLab, Northampton, MA, USA). Thermodynamic values are an average of at least 3 independent measurements.

### 4.11. Membrane Binding Assay

Urea-washed photoreceptor (rod outer segment) membranes were prepared from frozen retinae, as previously described [110]. The binding of NCS-1 and dNCS-1 to the membranes was analyzed by equilibrium centrifugation assay, as described in [10,111], under non-reducing conditions. Membrane-bound NCS-1 was visualized by Western blotting (see below) and weight fractions of the bound proteins were quantified by densitometric analysis of the bands using GelAnalyzer.2010a software (Istvan Lazar Jr., University of Budapest, Budapest, Hungary).

### 4.12. Rhodopsin Phosphorylation Assay

GRK1 activity in the presence of NCS-1 was assayed according to the previously described procedure with modifications [8,112]. Briefly, urea-washed photoreceptor membranes, containing dark-adapted rhodopsin (10 μM), were mixed with 5–60 µM NCS-1 or 2.5–30 µM dNCS-1, 1 mM [γ-^32^P]ATP and 2 μL (~0.16 μM) of recombinant GRK1. The reaction was performed in 20 mM Tris-HCl buffer (pH 8.0), 150 mM NaCl, 3 mM MgCl_2_ and 250 μM CaCl_2_. Rhodopsin phosphorylation was initiated by light illumination and terminated after 25 min by the addition of SDS-PAGE sample buffer. The proteins were separated by SDS-PAGE and γ-^32^P emission was registered by radioautography, using FLA-3000 PhosphorImager detection system (Fujifilm, Minato, Japan). Relative (as compared to NCS-1-free samples) effectiveness of rhodopsin phosphorylation was estimated from densitometrical analysis of the bands using GelAnalyzer.2010a software (Istvan Lazar Jr., University of Budapest, Budapest, Hungary).

### 4.13. Surface Plasmon Resonance (SPR) Spectroscopy

SPR measurements were performed at 25 °C using a Bio-Rad ProteOn™ XPR36 protein interaction array system and ProteOn GLH sensor chip (Bio-Rad, Hercules, CA, USA). Ligand (50 μg/mL GRK1^N-C^ in 10 mM sodium acetate buffer, pH 4.5) was immobilized on the chip surface (up to 12,000 RU) by amine coupling, according to the manufacturer’s instructions. The remaining activated amine groups on the chip surface were blocked by a 1 M ethanolamine solution. Analyte (NCS-1 or dNCS-1, 0.5 to 20 μM) in a running buffer (10 mM HEPES (pH 7.4), 150 mM NaCl, 1 mM CaCl_2_, 0.05% Tween 20) was passed over the chip at a rate of 30 μL/min for 350 s, followed by flushing the chip with the running buffer for 1200 s. The double-referenced SPR sensorgrams were globally fitted according to either a heterogeneous ligand model (assumes the existence of two populations of the ligand that bind single analyte molecule) for both monomers and dimers, or, alternatively, the bivalent analyte model (assumes that each analyte molecule binds two ligand molecules) for dNCS-1. Kinetic and equilibrium dissociation constant values were evaluated using Bio-Rad ProteOn Manager™ v.3.1 software (Bio-Rad, Hercules, CA, USA).

### 4.14. Cell Culture and Treatments

Experiments were performed using human embryonic kidney cells 293 (HEK293) and Y79 retinoblastoma cell lines. HEK293 cells were cultured in Dulbecco’s modified Eagle’s medium supplemented with 10% fetal bovine serum (FBS), 4 mM L-glutamine, 4.5 g/L D-glucose at 37 °C in a humidified atmosphere containing 5% CO_2_. Y79 cells were cultured in RPMI-1640 medium supplemented with 20% FBS, 4 mM L-glutamine, 4.5 g/L D-glucose at 37 °C in a humidified atmosphere containing 5% CO_2_. For NCS-1 transfection, HEK293 cells were cultured in six-well plates in the presence of 4 µg of NCS-1-pCI-neo plasmid and 4 µL of TurboFect transfection reagent for 3 h with subsequent medium replacement. Analysis of the transient NCS-1 expression by Western blotting and all experiments with HEK293 cells were performed 48 h after transfection.

For short-term incubations, ZnCl_2_ (50–2000 μM), chloroquine (300 μM), CaCl_2_ (3 mM), ionomycin (5 μM), EDTA (4 mM), EGTA (1 mM), and/or H_2_O_2_ (0.1–10 mM) were added directly into the HEK293 or Y79 cell medium. For recovery experiments, HEK293 cells were incubated with 10 mM H_2_O_2_ with or without preincubation in ZnCl_2_ (50–2000 μM), chloroquine (300 μM), CaCl_2_ (3 mM), ionomycin (5 μM), EDTA (4 mM), EGTA (1 mM), MG132 (20 μM), or auranofin (2 μM). The cells were cultivated for the indicated time intervals, harvested by thorough resuspension in the same medium (in the case of HEK293 cells, the medium was gently pipetted to ensure the detachment of all cells), and centrifuged at room temperature (RT). The pellets were resuspended in lysis buffer (50 mM Tris-HCl (pH 8.0), 100 mM NaCl, 0.5% NP-40) containing freshly added 10 mM iodoacetamide and EDTA-free protease inhibitor cocktail. To facilitate extraction, three freezing/thawing cycles of cell lysates at −80 °C/RT were performed. The obtained cell lysates were stored at −80 °C for further analysis.

### 4.15. Immunocytochemistry and Microscopy

HEK293 cells grown on glass coverslips in six-well plates were transfected with NCS-1-pCI-neo plasmid and treated as described above. All subsequent incubations were performed at room temperature using a plate shaker. For fixation, the cells were washed with PBS, incubated with 4% paraformaldehyde in PBS for 10 min, and washed with ice-cold PBS three times for 5 min. After that, the cells were permeabilized by incubation with 0.1% Triton X-100 in PBS for 10 min, washed three times with PBS, and blocked by incubation with 1% BSA in PBS with 0.1% Tween 20 (PBST) for 30 min. NCS-1 staining was performed by incubation of the cells with rabbit antibodies against NCS-1 [8] (in 1% BSA in PBST) for 1 h, washing three times in PBST, and incubation with goat anti-rabbit Alexa Fluor 555-conjugated IgG (in 1% BSA in PBST) for 1 h in the dark. For cytoskeleton actin staining, the cells were next washed three times in PBST and incubated with FITC-conjugated phalloidin (in PBS) for 30 min in the dark. Finally, the cells were washed three times in PBS and subjected to nuclear staining by incubation with DAPI in the dark for 2 min and washing with PBS. Coverslips with stained cells were mounted to a glass slide with a drop of mounting medium, allowed to solidify overnight at room temperature in the dark, and analyzed with an upright Axio Imager D2 microscope equipped with an Axiocam 506 mono camera using Zen 2.6 pro software (Zeiss, Oberkochen, Germany).

### 4.16. Analysis of Apoptosis by Flow Cytometry

NCS-1 silencing in Y79 cells was performed by their transfection with 60 pmol of NCS-1 siRNA and 6 µL of siRNA transfection reagent in FBS-free medium for 5 h with subsequent replacement with fresh medium with FBS and verified by Western blotting of cells harvested 24 h after the transfection. For apoptosis experiments, Y79 cells with or without NCS-1 siRNA transfection were incubated with 1 mM H_2_O_2_ for 6 h, collected, washed with Dulbecco’s phosphate-buffered saline without Ca^2+^ and Mg^2+^ (DPBS), and resuspended in annexin V-binding buffer. The cells were incubated with FITC-conjugated annexin V for 20 min in the dark chamber at room temperature, diluted with annexin V-binding buffer up to 400 mL, and analyzed using BD LSRFortessa flow cytometer (BD Biosciences, Franklin Lakes, NJ, USA) and BD FACSDiva software. Raw data analysis and visualization were performed in FlowJo software (BD Biosciences, Franklin Lakes, NJ, USA). 

### 4.17. Western Blotting

Western blotting of cell lysates and other probes was performed as described elsewhere [27]. Disulfide forms of NCS-1 were detected by performing SDS-PAGE under non-reducing conditions employing sample buffer without β-mercaptoethanol. The staining was conducted using rabbit polyclonal anti-NCS-1 antibodies obtained previously via immunization of rabbits with the recombinant protein [8]. The bands were visualized using the Enhanced Chemiluminescence reagent kit and ChemiDoc™ XRS+ gel documentation system (Bio-Rad, Hercules, CA, USA). For quantitative assessments, the samples were normalized by GAPDH expression and the amounts of NCS-1 forms were calculated by densitometric analysis of the bands using GelAnalyzer.2010a software (Istvan Lazar Jr., University of Budapest, Budapest, Hungary).

### 4.18. Molecular Modeling

Human Ca^2+^-free NCS-1 was built based on NMR structures of NCS-1 from *Schizosaccharomyces pombe* (PDB 2L2E) using MODELLER 10.1 (Laboratory of Andrej Sali, University of California, CA, USA) [113]. To reduce the number of the analyzed NMR structures, they were subjected to non-parametric clustering, resulting in the selection of two candidates as clusters centers (based on the 4th and 12th frames) [114]. Protein-protein docking of the NCS-1 variants was performed independently in ZDOCK 3.0.2 with a selection of poses characterized by the least distances between the sulfur atoms of cysteines and in HADDOCK 2.4 (Alexandre Bonvin research group, Utrecht University, Utrecht, The Netherlands), where cysteines were set as interface residues [115,116]. Final structures were further optimized using phenix.geometry_minimization from the Phenix 1.1.18 (Lawrence Berkeley National Laboratory, Berkeley, CA, USA) [117]. Dynamical signatures were computed using the ENCoM model as implemented in the NRGTEN 1.0.2 package (Najmanovich Research Group, University of Montreal, Montreal, QC, Canada) [118]. Visualizations of protein structures, interface contacts, and plots were created in the PyMOL 2.3.0 (Schrödinger, LLC., New York, NY, USA), LigPlot+, and Matplotlib, correspondingly [119,120].

### 4.19. Statistics

The data were collected from at least three independent experiments and analyzed using the mean standard error method. Statistical significance was assessed by a two-tailed Mann–Whitney U test, using Sigma Plot 12.0 (Systat Software, Chicago, IL, USA) data analysis toolkit. A *p*-value of less than 0.05 is considered significant.

## Figures and Tables

**Table 1:** Thermodynamic parameters of Ca^2+^ binding to dNCS-1, according to ITC data.

Protein	K_D_^1^, M	ΔH ^1^, kJ	K_D_^2^, M	ΔH ^2^, kJ	K_D_^3^, M	ΔH ^3^, kJ
NCS-1 ^1^	2.3 × 10^−7^	−10.1	5.0 × 10^−6^	1.4	2.9 × 10^−7^	−17.8
	**N ^1^**	**K_D_^1^, M**	**ΔH ^1^, kJ**	**N ^2^**	**K_D_^2^, M**	**ΔH ^2^, kJ**
dNCS-1 ^2,3^	2.1	(5.6 ± 0.8) × 10^−7^	−13.1 ± 0.2	-	-	-

^1^ Data from ref. [12] fitted using “Sequential binding (3 ions)” model. ^2^ Data fitted using “One set of sites” model. ^3^ The parameters are calculated per mole of dNCS-1.

**Table 2:** Kinetic parameters of NCS-1 and dNCS-1 binding to immobilized GRK1^N-C^, calculated from SPR spectroscopy data.

Analyte	k_on_^1^, s^−1^ M^−1^	k_off_^1^, s^−1^	K_D_^1^, M	k_on_^2^, s^−1^ M^−1^	k_off_^2^, s^−1^	K_D_^2^, M
NCS-1	267 ± 22	(1.6 ± 0.6) × 10^−4^	(5.9 ± 2.0) × 10^−7^	1910 ± 650	(4.4 ± 1.5) × 10^−3^	(2.7 ± 1.8) × 10^−6^
dNCS-1 ^1^	3580 ± 2230	(1.1 ± 0.2) × 10^−4^	(4.8 ± 3.9) × 10^−8^	1150 ± 350	(2.5 ± 0.3) × 10^−3^	(2.2 ± 0.5) × 10^−6^
dNCS-1 ^2^	9780 ± 5700	(2.3 ± 0.6) × 10^−4^	(3.0 ± 1.8) × 10^−8^	-	-	-

^1^ Data fitted using “Heterogeneous ligand” model. ^2^ Data fitted using “Bivalent analyte” model.

**Table 3:** Thermodynamic parameters of Zn^2+^ binding to dNCS-1, according to ITC data.

Interaction	N ^1^	K_D_^1^, M	ΔH ^1^, kJ	N ^2^	K_D_^2^, M	ΔH ^2^, kJ
NCS-1 ^1^	0.7	4.3 × 10^−6^	−11.8	2.0	1.1 × 10^−7^	−7.3
dNCS-1 ^2,3^	1.3	(4.7 ± 1.2) × 10^−7^	−3.9 ± 0.4	2.1	(1.2 ± 0.5) × 10^−8^	−6.6 ± 0.1

^1^ Data from ref. [12]. ^1,2^ Data fitted using “Two sets of sites” model. ^3^ The parameters are calculated per mole of dNCS-1.

## Supplementary Materials

The following are available online at www.mdpi.com/article/10.3390/ijms222212602/s1.
