# Supplementary material for: Disulfide Dimerization of Neuronal Calcium Sensor-1: Implications for Zinc and Redox Signaling
Source: Int J Mol Sci. 2021 Nov 22;22(22):12602. doi: 10.3390/ijms222212602 (PMC8623652; doi:10.3390/ijms222212602)
Supplement: Supplementary file 1 [file ijms-22-12602-s001.zip › ijms-1408907-supplementary.pdf]

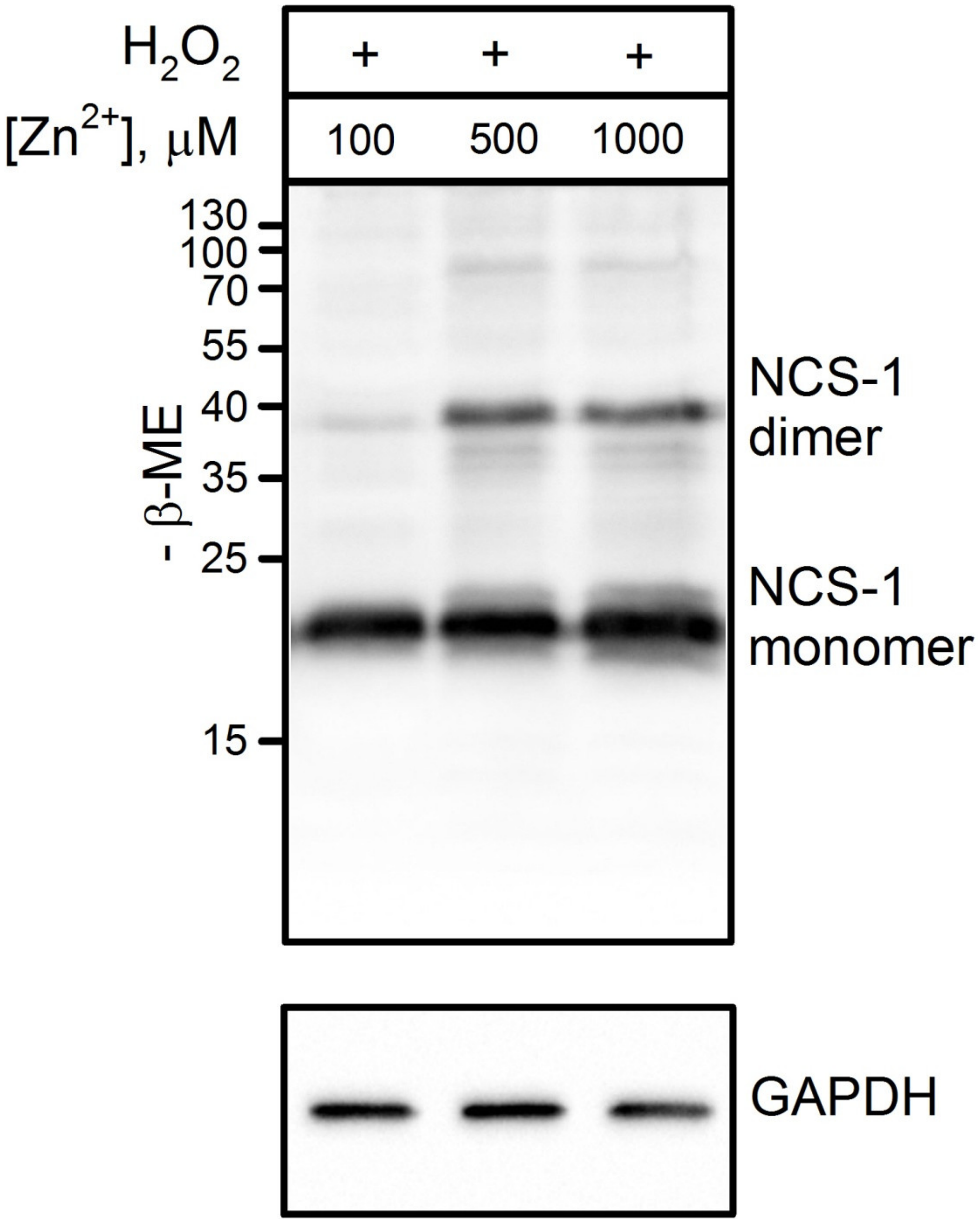

Figure S1. Zinc-dependent disulfide dimerization of NCS-1 in HEK293 cells. NCS-1-transfected cells were incubated for 60 min with 10 mM H_2O_2 and increasing concentrations of Zn^{2+} in the presence of zinc ionophore chloroquine. Disulfide forms of NCS-1 were detected by Western blotting of cell lysates conducted under non-reducing conditions.

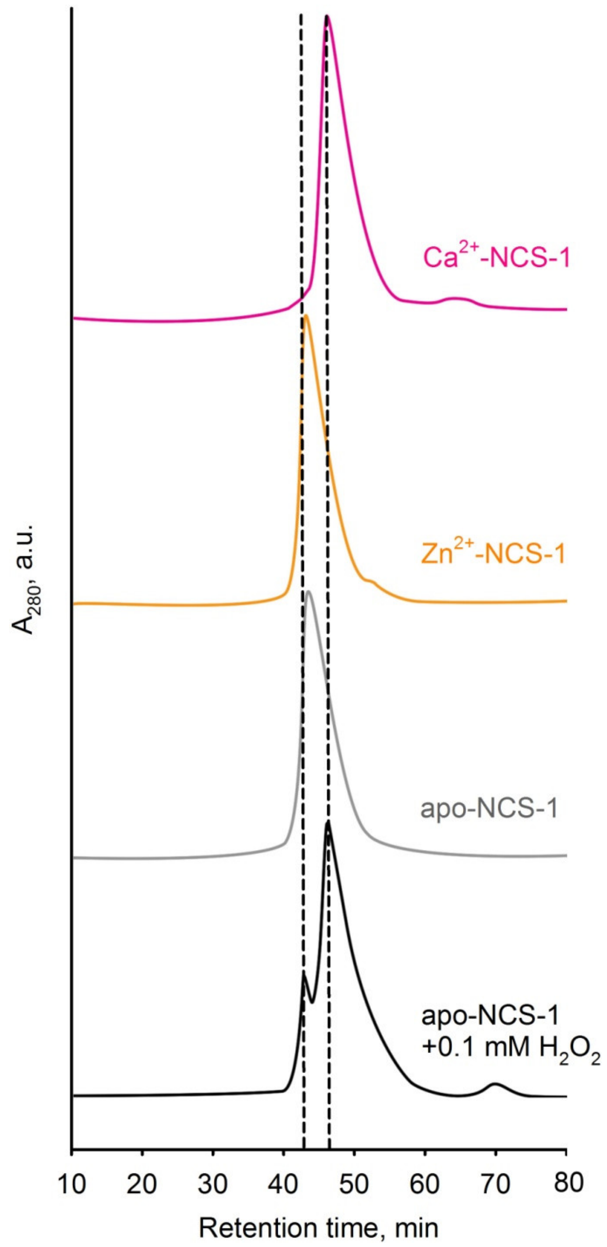

Figure S2. Zinc-dependent non-covalent dimerization of NCS-1 *in vitro*. Aliquots of recombinant NCS-1 were analyzed using high-resolution gel filtration in the presence of either 1 mM $CaCl_2$, 0.1 mM $ZnCl_2$ or 1 mM EGTA (apo-protein). Mixture of NCS-1 and dNCS-1 obtained as a result of brief incubation of the protein with 0.1 mM H_2O_2 was applied as reference.

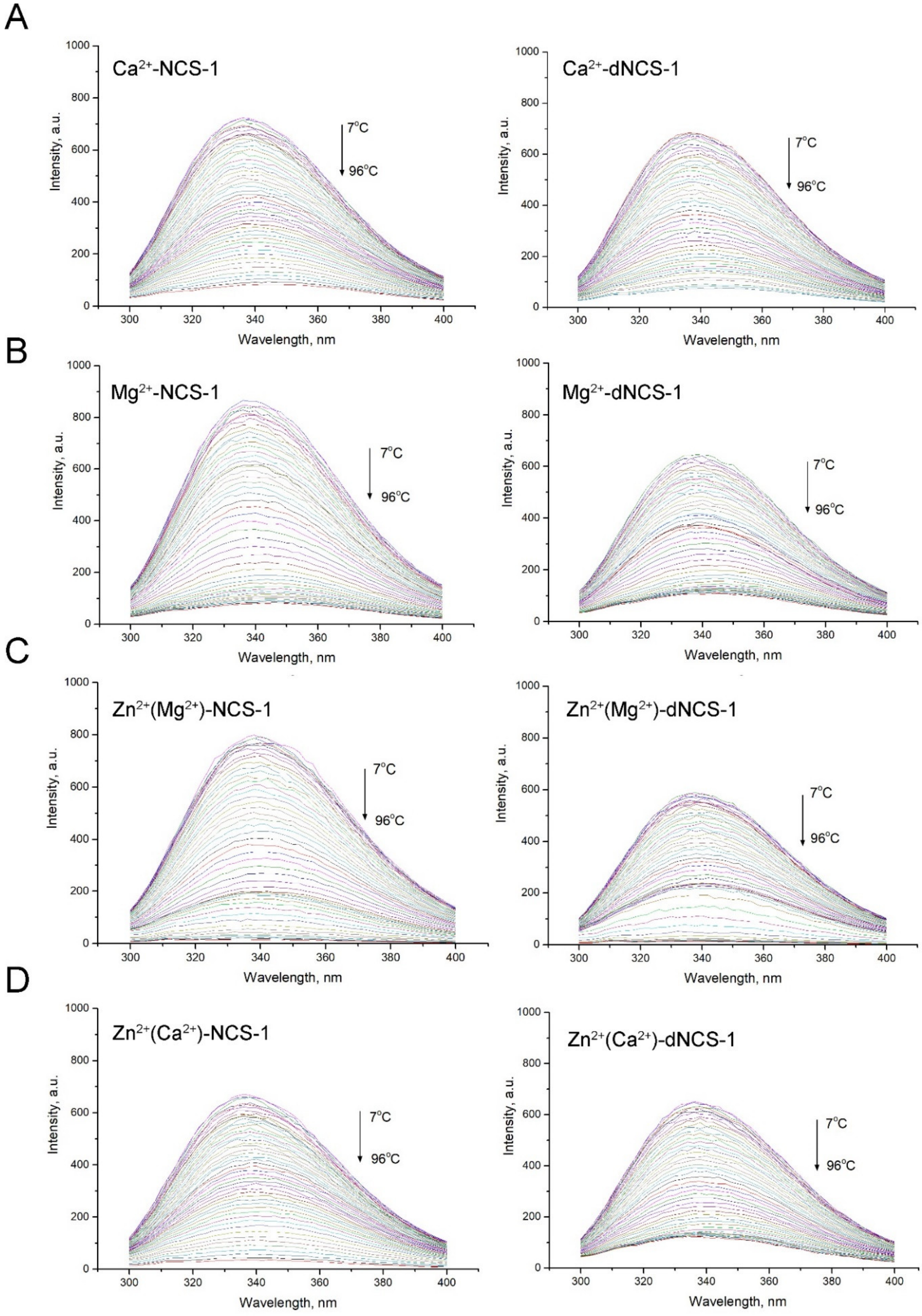

Figure S3. Monitoring of thermal denaturation of NCS-1 and dNCS-1 using intrinsic fluorescence measurements. Tryptophan fluorescence spectra of were registered at different temperatures (7-96 ºC) in the presence of 1 mM Ca^{2+} **(A)**, 1 mM Mg^{2+} **(B)**, 1 mM Mg^{2+} and 0.1 mM Zn^{2+} **(C)**, 1 mM Ca^{2+} and 0.1 mM Zn^{2+} **(D)**.
